# The Present Status of Antityphoid Inoculation

**Published:** 1911-03

**Authors:** 


### The Present Status of Antityphoid Inoculation

Gosman in the *Journal of the American Medical Association* of October 1, 1910, in a paper on this subject reaches the following conclusions: Considering all the data it seems that the present status of these inoculations against typhoid fever is that they are valuable as a method of preventing the disease and are perhaps the most valuable single asset we have in combating an epidemic, and there is no doubt in the minds of most thinking and up-to-date medical men that they should be used in the following classes of persons: first, all nurses, ward attendants, hospital corps men, Red Cross assistants, physicians, and medical students; also all persons who contemplate a journey into a section where typhoid is known to exist or is suspected of existing. Also, the inoculations should be done generally in districts suffering from an epidemic, and especially in the families where a case exists, and he adds that in time of war all volunteers at camps of concentration should be inoculated as soon as possible after the camp is started.

Finally, it seems that typhoid will always be with us, but there is no necessity of its always being with us in our armies to any such appalling extent as it has in the past; therefore, the necessity for antityphoid inoculations will surely always be with us, for no matter how careful we are of our sanitation, our milk, our water, our flies, our contact infection, we will always have one or more of our much-talked-of-friends, the chronic typhoid carriers, and it is only by inoculating them and other friends and acquaintances that we can keep more or less of them free from typhoid.

The author is convinced of the harmlessness, and at the same time of the effectiveness, of this procedure, and would recommend its universal adoption, to the end that our armies in times of peace and war, and our communities at all times, may be kept as free as possible from that terrible epidemic disease, typhoid fever.

And last, but not least, the author believes he can state from a careful study and investigation of the matter that the present status of antityphoid inoculation as a means of prevention is assured, and that its future possibilities are great.—*Therapeutic Gazette.*

